# The Effect of a 20 km Run on Appetite Regulation in Long Distance Runners

**DOI:** 10.3390/nu8110672

**Published:** 2016-10-26

**Authors:** Chihiro Kojima, Aya Ishibashi, Kumiko Ebi, Kazushige Goto

**Affiliations:** 1 Graduate School of Sport and Health Science, Ritsumeikan University, Kusatsu, Shiga 525-8577, Japan; sh0007ek@ed.ritsumei.ac.jp (C.K.); aya.ishibashi@jpnsport.go.jp (A.I.); ab@fc.ritsumei.ac.jp (K.E.); 2 Department of Sports Science, Japan Institute of Sports Science, Nishigaoka, Kitaku, Tokyo 115-0056, Japan

**Keywords:** appetite-related hormones, energy intake, long distance run, athletes

## Abstract

The purpose of the present study was to investigate appetite-related hormonal responses and energy intake after a 20 km run in trained long distance runners. Twenty-three male long-distance runners completed two trials: either an exercise trial consisting of a 20 km outdoor run (EX) or a control trial with an identical period of rest (CON). Blood samples were collected to determine plasma acylated ghrelin, peptide YY_3-36_ (PYY_3-36_) and other hormonal and metabolite concentrations. Energy intake during a buffet test meal was also measured 30 min after the exercise or rest periods. Although plasma acylated ghrelin concentrations were significantly decreased after the 20 km run (*p* < 0.05), plasma PYY_3-36_ did not change significantly following exercise. Absolute energy intake during the buffet test meal in EX (1325 ± 55 kcal) was significantly lower than that in CON (1529 ± 55 kcal), and there was a relatively large degree of individual variability for exercise-induced changes in energy intake (−40.2% to 12.8%). However, exercise-induced changes in energy intake were not associated with plasma acylated ghrelin or PYY_3-36_ responses. The results demonstrated that a 20 km run significantly decreased plasma acylated ghrelin concentrations and absolute energy intake among well-trained long distance runners.

## 1. Introduction

Appetite regulation is closely associated with circulating hormones secreted from digestive organs. Plasma ghrelin, secreted from the stomach, is known to be the only hormone that promotes hunger and food intake [1]. In contrast, peptide YY_3-36_ (PYY_3-36_) and glucagon-like peptide-1 (GLP-1) are produced in the gastrointestinal tract. These hormones have the opposite role of ghrelin, resulting in attenuation of appetite [2,3,4,5]. Recently, attention to the influence of acute exercise on feeding behavior and its related endocrine regulations has increased. King et al. [6] showed that plasma ghrelin concentrations and subjective feelings of hunger were significantly impaired by 90 min of running at 70% of maximal oxygen uptake ($\dot{V}$O_2max_). Moreover, Martins et al. [7] demonstrated that plasma GLP-1 and PYY_3-36_ concentrations were significantly increased by 60 min of endurance exercise at 65% of $\dot{V}$O_2max_. Jokisch et al. [8] suggested that energy intake during a buffet test meal was reduced significantly after exercise compared with rest conditions for sedentary males. Although some inconsistent results still exist [9,10], the attenuating effect of exercise on hunger and energy intake is well established [6,7,11,12]. These findings could contribute to the design of optimal exercise prescriptions for health promotion and protection against obesity.

In contrast to the abundant studies on untrained individuals, appetite regulation after high-intensity (above 80% of $\dot{V}$O_2max_) and prolonged (>60 min) exercise, which is commonly incorporated into the daily training program of trained athletes, remains under exploration. Sim et al. [13] showed that high-intensity interval training (HIIT, 15 s sprint at 170% of $\dot{V}$O_2max_ with 60 s active recovery at 32% of $\dot{V}$O_2max_) suppressed subsequent ad libitum energy intake and ghrelin concentrations in obese individuals. Deighton et al. [14] investigated the influence of HIIT (30 s all-out sprint with 4 min active recovery at 30 W) on appetite regulation in young untrained males, with the results suggesting that subjective feelings of appetite, as well as ghrelin concentrations, were markedly suppressed following HIIT. However, previous studies which investigated the effects of prolonged exercise (>60 min) on appetite regulation are limited. In particular, the majority of previous studies were conducted in a laboratory setting. To our knowledge, no study found any influence from prolonged exercise among well-trained athletes during actual training in the field. Since trained athletes experience greater exercise-induced metabolic and endocrine responses compared with individuals with lower fitness levels [15], their levels of exercise-induced appetite suppression may be more profound. Reduction of energy intake by exercise (exercise-induced anorexia) may be beneficial for weight management. However, athletes are commonly required to facilitate recovery of energy substrates (e.g., muscle glycogen, intramyocellular lipid) and promote muscle protein synthesis after training. Impaired energy intake after strenuous exercise is thought to delay recovery of exercise capacity and to promote accumulated fatigue. Levenhagent et al. [16] demonstrated that nutrient intake immediately after exercise enhanced glucose uptake and protein synthesis in the leg and whole body muscles when compared with consuming the same meal 3 h after exercise. Considering the importance of nutrient intake during the early phase of the post-exercise period, elucidation of appetite regulation during the early phase of prolonged high-intensity exercise in athletes is valuable.

In the present study, we investigated the time course of changes in appetite-related hormonal responses and spontaneous energy intake after a 20 km outdoor run (approximately 78 min in duration) in trained long distance runners. We hypothesized that the run would result in decreased spontaneous energy intake during a subsequent meal, with lowered plasma acylated ghrelin concentrations and elevated plasma PYY_3-36_ concentrations.

## 2. Materials and Methods

### 2.1. Subjects

Twenty-three male, college endurance runners (age, 20.0 ± 0.3 (mean ± standard error) years; height, 171.2 ± 1.9 cm; weight, 56.3 ± 1.0 kg; BMI, 19.3 ± 0.4 kg/m^2^; and $\dot{V}$O_2max_, 67.1 ± 1.0 mL/kg/min) participated in this study. All subjects belonged to the same running team, which specialized in long-distance running and maintained regular practice (2.5 h/day) 6 times a week. Subjects were informed of the purpose, experimental procedures, and risks of the study, and written informed consent was obtained from all participants. The study was approved by the Ethics Committee for Human Experiments of Ritsumeikan University (BKC-IRB-2014-015), Japan.

### 2.2. Experimental Design

Prior to conducting experiments, $\dot{V}$O_2max_ was determined using incremental running test. Subjects started running at 14 km/h and running velocity was increased by 2 km/h every 4 min until 18 km/h. Once the running velocity reached 18 km/h, it was increased by 0.6 km/h every 1 min until exhaustion. Respiratory gases were collected and analyzed using an automatic gas analyzer (AE310S, Minato Medical Science Co., Ltd., Tokyo, Japan). The collected data were averaged every 30 s.

All subjects completed two trials on different days. The first visit was designed as an exercise trial (EX), and the second visit consisted of a trial without exercise (CON). Each trial was separated by 1 week. Due to experimental setting with performing 20 km outdoor run, the present study was conducted without crossover-design to match environmental factor within subjects during a 20 km run. We selected a 20 km run because it was actually incorporated into the training program in long distance runners. Exercise-induced metabolic and hormonal responses, subjective appetite, and energy intake after exercise or rest were compared between the two trials. On the day prior to the trials, the content of regular practice and calories consumed during dinner were matched to avoid any influence on metabolic and appetitive responses on the following day. Dinner was provided between 8:00 p.m. and 9:00 p.m. and consisted of regular Japanese food. The total calories consumed (1331 ± 50 kcal) were identical in each trial. Subjects stayed in accommodations at the university, and their scheduled sleep time was set from 11:00 p.m. to 6:30 a.m.

On the measurement days, the subjects arrived at the laboratory at 7:00 a.m. following an overnight fast, and they rested for at least 20 min before blood collection. On the EX day, all subjects completed a 20 km outdoor run between 7:30 a.m. and 10:30 a.m. They were instructed to run at a prescribed pace and their elapsed time was monitored. Heart rate was recorded continuously every 15 s during exercise using a heart rate monitor (Polar RCX5, Polar Electro Oy, Kempele, Finland). Subjects were allowed to consume a total of 400 mL of water during the exercise period. The ambient temperature during the run was 12.1 °C. On the CON day, the subjects did not engage in exercise, and instead rested in the laboratory for a period of time identical to that taken to complete the run. During this period, they were allowed to read books and were required to consume the same amount of water (400 mL) they had consumed on the day of EX. The room temperature was set at 19 °C.

Blood sampling, evaluation of subjective feelings of appetite using a visual analog scale (VAS), and respiratory gas sampling were conducted several times before and after exercise or rest, and 30 min following the 20 km outdoor run or rest period. Thirty min after the run or the rest period, energy and macronutrient intake during a buffet test meal were evaluated.

### 2.3. Blood Parameters

On the experimental trial days, subjects arrived at the laboratory at 7:00 a.m. following an overnight fast. After resting for 20 min, a baseline blood sample was obtained. A series of blood samples were subsequently collected immediately after exercise or rest, and 30 min following the exercise or rest period. Serum and plasma samples were obtained by centrifugation (10 min, 4 °C) and stored at −80 °C until analysis. From the obtained samples, plasma acylated ghrelin and PYY_3-36_, serum growth hormone (GH), free fatty acids (FFA), creatine kinase (CK), and myoglobin (Mb) concentrations were measured. Blood glucose and lactate concentrations were measured immediately after blood collection using a glucose analyzer (Free Style, Nipro Co., Osaka, Japan) and a lactate analyzer (Lactate Pro, ARKRAY Co., Kyoto, Japan), respectively. Blood glucose measurements were performed in duplicate and the average values were used. Serum GH concentration was measured using electrochemiluminescence immunoassay. Serum FFA concentration was measured using enzymatic methods. Serum CK and Mb concentrations were measured at a clinical laboratory (SRL Inc., Tokyo, Japan). The intra-assay coefficients of variation (CV) were 1.9% for GH, 1.3% for FFA, 2.8% for CK, and 2.4% for Mb.

For the measurement of plasma acylated ghrelin and PYY_3-36_ concentrations, blood was drawn into a chilled tube containing EDTA, dipeptidyl peptidase-4 (DPP-IV), protease, and esterase inhibitors. After obtaining plasma by centrifugation at 4 °C, hydrochloric acid (1 mmol/L) was immediately added to the micro tube for acylated ghrelin analysis, following the manufacturer’s instructions. Plasma acylated ghrelin concentration was measured using an enzyme-linked immunosorbent assay (ELISA) kit (Mitsubishi Chemical Medicine Corp., Tokyo, Japan). The intra-assay CV was 4.6%. The plasma PYY_3-36_ concentration was measured using an ELISA kit (Phoenix Pharmaceuticals, Inc., Burlingame, CA, USA) and the intra-assay CV was 6.1%. All ELISAs were performed in duplicate.

### 2.4. Subjective Feelings of Hunger, Appetite, Perceived Food Consumption, Satiety, and Fatigue

Ratings of subjective hunger, appetite, perceived food consumption, satiety, and fatigue were evaluated using a 100 mm VAS [17] before exercise (or rest), immediately after exercise (or rest), at 15 and 30 min after exercise (or rest), and after the buffet test meal.

### 2.5. Respiratory Parameters

A resting expired gas sample was collected 20 min after completing the 20 km run or rest. The subjects sat on a comfortable chair, and a respiratory gas sample was collected for 3 min and analyzed using an automatic gas analyzer (AE310S, Minato Medical Science Co., Ltd., Tokyo, Japan) to evaluate oxygen uptake ($\dot{V}$O_2_), carbon dioxide output ($\dot{V}$CO_2_), ventilatory volume ($\dot{V}E$), and the respiratory exchange ratio (RER). The values were averaged every 30 s. Appropriate calibrations of O_2_ and CO_2_ sensors and the volume transducer were performed using calibration gases and a 2 L syringe immediately before measurements were taken.

### 2.6. Ad Libitum Buffet Meal

A buffet test meal was started 30 min after the 20 km run or rest to evaluate energy and macronutrient intake. The meal lasted 30 min; however, the participants were not informed of the elapsed time during the test. All subjects were instructed to “eat until they felt comfortable satiety” in a separate environment from other subjects. The buffet meal consisted of abundant food items eaten regularly in standard Japanese breakfasts and included rice balls, bread, jam, grilled salmon, boiled beef, ham, sausages, boiled eggs, potato salad, natto (fermented soybean), boiled spinach, miso soup, milk, yogurt, cheese, oranges, apples, bananas, green tea, orange juice, and vegetable juice. The energy intake was determined by counting number of plates (calorie for each plate is already known) and by weighting remaining foods after eating. A dietary analysis program (Excel Eiyou-kun version 6.0, Kenpakusha, Tokyo, Japan) was also used to calculate energy intake and macronutrient content.

### 2.7. Statistical Analysis

Data are expressed as means ± SE. For all variables, normal distribution was confirmed using Kolmogorov-Smirnov test. Time courses of changes in blood parameters and subjective feelings of appetite were compared using a two-way repeated-measures analysis of variance (ANOVA) to determine interaction (trial × time) and main effects (trial, time). When ANOVA revealed a significant interaction or main effect, a Tukey-Kramer post hoc test was performed. Energy intake and respiratory gas parameters were compared between the two conditions using a paired *t*-test. The relationship between the exercise-induced relative change in energy intake and each blood parameter was determined using Pearson correlation coefficients. Statistical significance was accepted as a *p*-value < 0.05.

## 3. Results

### 3.1. Exercise Duration and Heart Rate Response during the 20 km Run

The average time taken to complete the 20 km run was 77.9 ± 0.3 min. The average heart rate (HR) during exercise was 157 ± 3 beats/min. The estimated percentage for maximum HR was 78.0% ± 1.3%.

### 3.2. Scores for Subjective Appetite and Fatigue

Table 1 shows the time-course changes in subjective scores for appetite and fatigue. A significant interaction (trial × time), as well as main effects of trial and time, were observed for hunger and appetite (*p* < 0.05). Hunger scores were significantly lower in EX than in CON immediately and 15 min after exercise (*p* < 0.05); however, this significant difference was not observed between the trials 30 min after exercise. Similarly, scores of appetite were significantly lower in EX compared with CON immediately and 15 min after exercise (*p* < 0.05). A two-way ANOVA revealed a significant interaction (trial × time) effect, and a main effect of time for perceived food consumption (*p* < 0.05). Perceived food consumption was significantly lower in EX compared with CON immediately after exercise (*p* < 0.05). Significant main effects of trial and time for satiety were observed (*p* < 0.05). Satiety scores were significantly higher in EX than in CON immediately, 15 min and 30 min after the exercise period (*p* < 0.05). Two-way ANOVA revealed a significant interaction effect (trial × time), as well as main effects of trial and time, for fatigue. In EX, scores for fatigue were significantly increased immediately and 15 min after exercise (*p* < 0.05). In addition, fatigue scores were significantly higher in EX than in CON at all time points after the exercise period (*p* < 0.05).

### 3.3. Blood Parameters

Table 2 shows the time-course of changes in blood glucose, lactate, serum GH, FFA, Mb, and CK concentrations. No significant differences between the trials were observed at baseline (before exercise or rest) for any blood parameters, expect for blood glucose concentrations. A significant interaction (trial × time) and a main effect of time were observed. Blood glucose concentrations were significantly increased immediately after the exercise period compared with CON (*p* < 0.05). However, blood glucose concentrations were significantly lower in EX compared with those in CON 30 min after exercise (*p* < 0.05). No significant interaction (trial × time), or main effects of time or trial were observed for blood lactate concentrations (*p* < 0.05). Blood lactate concentrations did not significantly change from baseline values in either trial. Two-way ANOVA revealed a significant interaction (trial × time), as well as main effects of time and trial, for serum GH, FFA, and Mb concentrations. Serum GH concentrations were significantly increased after exercise in EX (*p* < 0.05). Thirty min after exercise, serum GH concentrations remained significantly higher in EX compared with CON (*p* < 0.05). Serum FFA concentrations were markedly increased after exercise (*p* < 0.05), and were significantly different to those in CON (*p* < 0.05). Serum Mb concentrations were significantly increased immediately and 30 min after exercise (*p* < 0.05), and were also significantly different between EX and CON (*p* < 0.05). Lastly, a significant interaction (trial × time) as well as main effects of time for serum CK concentrations were observed. Although serum CK increased significantly with exercise (*p* < 0.05), there was no significant difference between the trials at any point (main effect of trial; *p* > 0.05).

**Figure 1:**
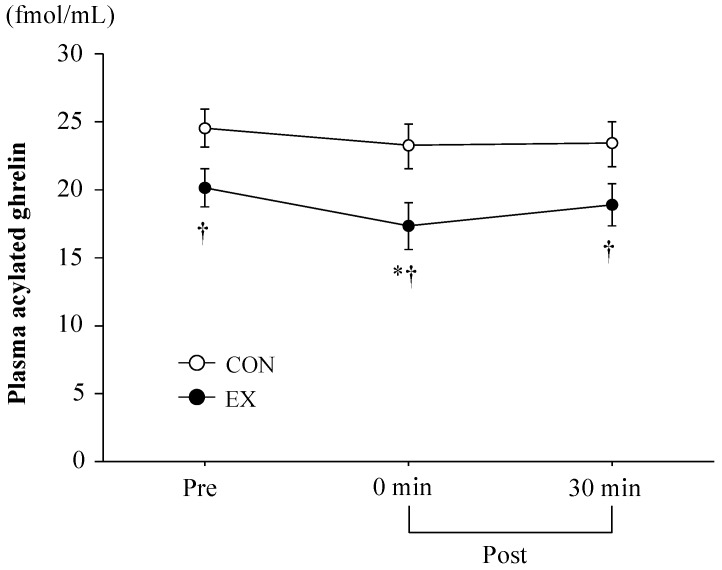
Change in plasma acylated ghrelin concentrations. Values are means ± SE. * *p* < 0.05 vs. pre, ^†^ *p* < 0.05 vs. CON.

Figure 1 shows the changes in plasma acylated ghrelin concentrations. Significant main effects of time and trial were observed for plasma acylated ghrelin (*p* < 0.05). Plasma acylated ghrelin concentrations at baseline were significantly lower in EX compared with CON (*p* < 0.05) and exercise significantly decreased plasma acylated ghrelin concentrations immediately after the exercise period (before exercise, 20.2 ± 1.4 fmol/mL; immediately after exercise, 17.3 ± 1.7 fmol/mL, *p* < 0.05) with a significant reduction relative to CON (*p* < 0.05). Thirty minutes after exercise, plasma acylated ghrelin concentrations remained significantly lower in EX than in CON (*p* < 0.05). In contrast, the CON trial did not show significant change in acylated ghrelin concentration over time.

**Figure 2:**
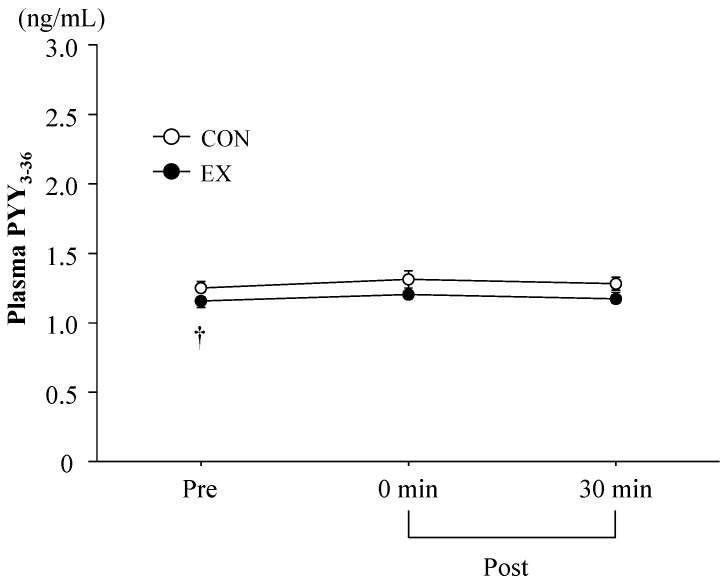
Change in plasma PYY_3-36_ concentrations. Values are means ± SE. ^†^ *p* < 0.05 vs. CON.

Figure 2 shows the time-course change of plasma PYY_3-36_ concentrations. Two-way ANOVA revealed a significant main effect of the trial for plasma PYY_3-36_ concentration. Although plasma PYY_3-36_ concentrations at baseline were significantly lower in EX than in CON (*p* < 0.05), there was no significant difference between the trials after exercise. Furthermore, plasma PYY_3-36_ concentration did not change significantly from baseline values in either EX or CON.

### 3.4. Respiratory Parameters

Resting $\dot{V}$O_2_ and $\dot{V}$CO_2_ after exercise were significantly higher in EX than in CON ($\dot{V}$O_2_, 271 ± 5 mL/min in EX vs. 233 ± 8 mL/min in CON; $\dot{V}$CO_2_, 202 ± 6 mL/min in EX vs. 182 ± 6 mL/min in CON, *p* < 0.05). Moreover, there was a trend toward lower RER in EX than in CON (0.75 ± 0.02 in EX vs. 0.78 ± 0.02 in CON, *p* = 0.056) and toward higher $\dot{V}E$ in EX than in CON (8.7 ± 0.4 L/min in EX vs. 7.9 ± 0.3 L/min in CON, *p* = 0.057).

### 3.5. Energy and Macronutrient Intake

Table 3 shows the energy intake, macronutrient intake ratios, and types of menu selected during the buffet test meal. The time required to finish eating was not significantly different between EX and CON. Energy intake was significantly lower in EX (1325 ± 55 kcal) compared to CON (1529 ± 55 kcal, *p* < 0.05); the exercise-induced relative change in energy intake was −12.9% ± 2.8%. With regard to macronutrient distribution, fat intake was significantly lower in EX than in CON (*p* < 0.05), while carbohydrate intake was significantly higher in EX than in CON (*p* < 0.05). Moreover, comparing the caloric intake within four categories of food (staple foods, others, fruits, and drinks) among the 21 different menus indicated that calories consumed from staple foods, including carbohydrates (e.g., rice, bread) and others (e.g., fish, meat) were significantly lower in EX (*p* < 0.05). In contrast, calories consumed from drinks including tea, juice, milk, and soups were slightly but significantly greater in EX (*p* < 0.05).

### 3.6. Inter-Individual Variability in Exercise-Induced Changes in Energy Intake

**Figure 3:**
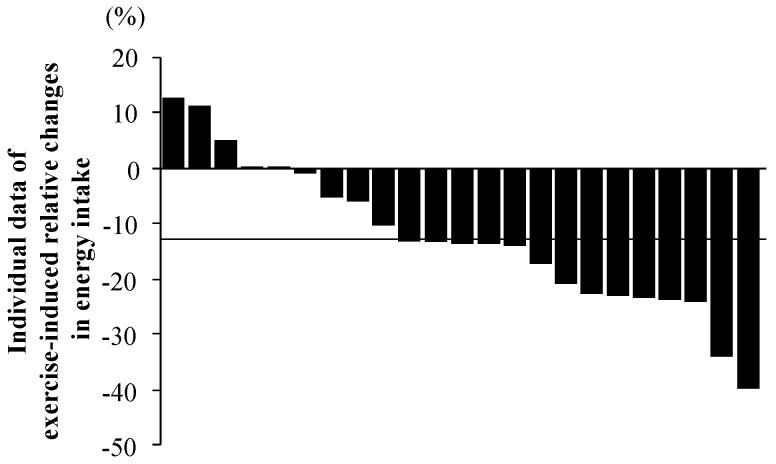
Individual data of exercise-induced relative change in energy intake. The line indicates the average value of relative change in exercise-induced energy intake (−12.9% ± 2.8%).

Figure 3 shows the individual data of exercise-induced relative changes in energy intake [(energy intake in EX − energy intake in CON)/energy intake in CON × 100]. A relatively large individual difference in energy intake was observed (ranging from −40.2% to 12.8%). In total, 3 of 23 subjects (13.0%) had increased energy intake after the exercise period compared with rest, while there were no differences in intake between the trials in 2 subjects (8.7%). However, 18 subjects (78.3%) had reduced energy intake after exercise compared with rest.

### 3.7. Correlation between Exercise-Induced Relative Changes in Energy Intake and Blood Variables

When the relationship between the exercise-induced relative change in energy intake and blood variables was determined, changes in energy intake showed a significant inverse correlation with serum Mb concentrations 30 min (*r* = −0.477, *p* < 0.05) after exercise. Moreover, there was an inverse trend of correlation between exercise-induced relative change in energy intake and serum Mb concentrations immediately after exercise (*r* = −0.372, *p* = 0.08). Exercise-induced absolute change in energy intake showed an inverse trend of correlation with that in area under the curve (AUC) of serum Mb (*r* = −0.393, *p* = 0.06). However, plasma acylated ghrelin concentrations immediately (*r* = 0.11, *p* = 0.61) and 30 min (*r* = 0.20, *p* = 0.37) after exercise did not correlate significantly with exercise-induced relative changes in energy intake. Similarly, plasma PYY_3-36_ concentrations immediately (*r* = 0.12, *p* = 0.58) and 30 min (*r* = 0.03, *p* = 0.90) after exercise were not correlated with exercise-induced relative changes in energy intake. No significant relationship was observed between exercise-induced absolute change in energy intake and AUCs of ghrelin (*r* = −0.05, *p* = 0.81) or PYY_3-36_ (*r* = −0.34, *p* = 0.11).

## 4. Discussion

The present study was designed to determine the impact of a 20 km run on appetite regulation in well-trained long distance runners. We found that absolute energy intake during a buffet test meal after the exercise period was significantly reduced compared with that after a rest period of identical duration. However, this reduction in energy intake was not associated with exercise-induced acylated ghrelin or PYY_3-36_ responses.

### 4.1. Exercise-Induced Ghrelin and PYY_3-36_ Responses

Plasma acylated ghrelin concentrations were significantly decreased after exercise, in agreement with previous studies [6,18,19]; however, the magnitude of the reduction in plasma acylated ghrelin concentrations (14% reduction) was smaller compared to previously reported values [14,18,20]. A relatively small reduction in plasma acylated ghrelin could be due to a moderate GH response, as exercise-induced GH elevations suppress ghrelin [21,22,23]. In the present study, because the magnitude of the exercise-induced GH response was modest (pre-exercise, 1.8 ± 0.5 ng/mL; immediately after exercise, 8.9 ± 1.8 ng/mL), it may have resulted in a relatively small ghrelin response. Furthermore, plasma PYY_3-36_ concentrations were not significantly elevated after the exercise period. There have been inconsistencies in the reported effect of acute exercise on PYY_3-36_ response [12,13,14,24]. These inconsistencies may be due to whether breakfast was consumed prior to exercising or not. In fact, two studies that demonstrated exercise-induced PYY_3-36_ elevations provided a standard breakfast before the exercise period [12,14], while exercise in the present study was completed following an overnight fast.

### 4.2. Energy Intake Following 20 km Run

The most important finding in the present study was a significant reduction in energy intake after a 20 km run in well-trained long distance runners. In addition, a detailed analysis of the selected menus during the buffet test meal indicated that lowered energy intake in EX was due to a reduction in calories consumed from staple foods (e.g., rice and bread) and other foods (e.g., fish or meat), and not from fruits or drinks. Previous reports investigating the effect of acute exercise on appetite regulation among well-trained athletes ($\dot{V}$O_2max_ above 65 mL/kg/min) are quite limited. Moreover, this is the first study, to our knowledge, demonstrating that acute exercise decreased absolute energy intake in well-trained athletes. Exercise-induced decreases in appetite have been generally accepted to be dependent on exercise intensity [25]. However, the average HR (157 ± 3 bpm) during the 20 km run did not reach a maximal level (78.0% ± 1.3% of estimated maximal HR). Moreover, blood lactate concentrations did not change significantly from baseline, suggesting that exercise intensity was moderate. Resting $\dot{V}$O_2_, which was determined after the 20 km run, was significantly elevated 20 min after the exercise period, and resting energy expenditure was increased during the post-exercise period. Therefore, reduction of absolute energy intake with a concomitant increase in resting energy expenditure might promote a negative energy balance. However, caution is necessary since energy expenditure during a 20 km run was not evaluated in the present study. Further investigations need to determine energy expenditure during exercise and post-exercise for confirming energy availability. In addition, the influence of low energy availability on post-exercise recovery and training adaptations is required to explore.

### 4.3. Inter-Individual Variability of Exercise-Induced Reduction of Energy Intake

The relatively larger sample size of this study (*n* = 23) enabled us to perform additional statistical analyses. Although energy intake in EX was significantly lower than in CON, a relatively large degree of individual variability was observed (−40.2% to 12.8%); a similar trend was also reported in previous studies [24,26,27,28]. To clarify the association between appetite-related hormonal responses and exercise-induced reductions in energy intake, we divided all subjects into two groups (a group with greater reductions in energy intake and a group with smaller reductions in energy intake) based on the average value of the exercise-induced relative change in energy intake (−12.9% ± 2.8%). There were no significant differences between the two groups for acylated ghrelin (*p* = 0.78) or PYY_3-36_ (*p* = 0.53) concentrations 30 min after the exercise period (immediately before the buffet test meal), suggesting that reduced energy intake after a 20 km run was not associated with exercise-induced acylated ghrelin or PYY_3-36_ responses. Thus, a plausible factor contributing to the reduction in energy intake after the 20 km run may be an exercise-induced elevation of GLP-1, as GLP-1 has anorexigenic effects. Ellingsgaard et al. [29] suggested that GLP-1 secretion was stimulated by exercise-induced interleukin-6 (IL-6) production in skeletal muscle in rats. IL-6 is an inflammatory cytokine, and long distance running markedly increases IL-6 production in skeletal muscle, as well as in the blood [30]. Ueda et al. [12] also observed a significant inverse correlation between the decrease in energy intake after exercise and an incremental GLP-1 response. Therefore, the impact of GLP-1 concentration mediated by IL-6 elevation after a 20 km run on reduced energy intake should be considered. Furthermore, we found that exercise-induced elevations of Mb concentrations were significantly correlated with exercise-induced relative changes in energy intake. Currently, we are unsure whether increased Mb concentrations directly affected exercise-induced reductions in energy intake. However, future investigations to study the influence of muscle damage and the inflammatory response on appetite regulation would be informative. Another possible factor contributing to the reduction of energy intake might be an exercise-induced elevation of core temperature, since this has been demonstrated to lower energy intake after exercise [31]. However, it is unlikely that an elevation of core temperature had a strong impact in our study, since the 20 km run was completed in a cold environment during winter (ambient temperature: 12.1 °C).

### 4.4. Macronutrient Intake Following a 20 km Run

The influence of exercise on macronutrient intake distribution remains unclear [10,32,33]. Blundell et al. [34] suggested that acute exercise may alter food preference, which is associated with replenishment of short-term energy stores. In the present study, carbohydrate intake during the buffet test meal was significantly higher in EX than in CON. Considering that fat oxidation (evaluated by RER) was significantly enhanced during the post-exercise period in EX, replenishment of muscle glycogen appeared to be augmented after the 20 km run. Therefore, the increased proportion of carbohydrate is reasonable. Dehydration and thirst may have also contributed to altered macronutrient intake [33]. However, it is unlikely that dehydration occurred since the run was performed in a cold outdoor environment, and a total of 400 mL of water was provided to each participant. Furthermore, body weight did not change significantly after the exercise period (pre: 56.3 ± 1.0 kg, post: 55.8 ± 1.0 kg, *p* > 0.05).

### 4.5. Limitations

Several limitations should be considered in the present study. First, there were slight, but significant differences in baseline acylated ghrelin and PYY_3-36_ concentrations between the two trials. The reason for this difference is unclear as training volume, dinner before the testing day, and accommodations the night prior to the trial were controlled and matched between the two trials. However, there was no significant correlation between energy intake and acylated ghrelin or PYY_3-36_ concentrations at baseline (acylated ghrelin, EX: *r* = −0.16, *p* = 0.47, CON: *r* = −0.16, *p* = 0.47; PYY_3-36_, EX: *r* = −0.35, *p* = 0.10, CON: *r* = −0.17, *p* = 0.45). In addition, in the EX trial, the lower acylated ghrelin concentration (anorexigenic effect) might be offset by the lower PYY_3-36_ concentration (orexigenic effect). Therefore, the influence of different acylated ghrelin and PYY_3-36_ concentrations at baseline would have been negligible; Second, the present study was conducted without using randomized counter-balanced design to match environmental condition during 20 km outdoor run, and all subjects completed firstly EX. Unick et al. [35] revealed that energy intake during the meal was similar in spite of the order of exercise trial and rest trial (exercise trial followed by rest trial or rest trial followed by exercise trial). Therefore, it is assumed that the present experimental design without crossover design had little influence on energy intake during buffet test meal; Third, exercise intensity during the 20 km run may have been lower than during a competition. Therefore, the reduction of energy intake after exercise may have been underestimated. Finally, information on energy intake during the rest of the trial day, and on following day, was not recorded. Previous studies reported that a compensatory increase in energy intake after an ad libitum meal was not observed over such a time period [6,13,36]. However, caution is necessary because subjects of above studies [6,13,36] were not competitive endurance athletes. Further investigations are needed to confirm whether compensatory increase in energy intake will happen.

## 5. Conclusions

A 20 km run significantly decreased subjective hunger, plasma acylated ghrelin concentrations, and absolute energy intake in well-trained long distance runners. However, the exercise-induced reduction of energy intake was not associated with acylated ghrelin or PYY_3-36_ responses.

## Figures and Tables

**Table 1:** Change in scores of subjective feeling of appetite and fatigue.

		Pre	Post			After Meal
			0 min	15 min	30 min	
Hunger (mm)	EX	57 ± 4	51 ± 6 ^†^	60 ± 5 ^†^	68 ± 4	11 ± 1 *
	CON	60 ± 3	70 ± 3 *	71 ± 3 *	72 ± 3 *	16 ± 3 *
Appetite (mm)	EX	59 ± 5	52 ± 7 ^†^	62 ± 5 ^†^	68 ± 5	18 ± 4 *
	CON	58 ± 4	70 ± 3 *	72 ± 3 *	72 ± 3 *	23 ± 4 *
Prospective food consumption (mm)	EX	63 ± 4	54 ± 6 ^†^	61 ± 5	69 ± 4 *	20 ± 4 *
	CON	58 ± 4	67 ± 3 *	70 ± 2 *	68 ± 3	22 ±3 *
Satiety (mm)	EX	30 ± 4	29 ± 5 ^†^	32 ± 5 ^†^	31 ± 5 ^†^	82 ± 4 *^,†^
	CON	26 ± 3	20 ± 3	18 ± 5	18 ± 3	70 ± 5 *
Fatigue (mm)	EX	43 ± 4 ^†^	58 ± 4 *^,†^	55 ± 4 *^,†^	52 ± 4 ^†^	41 ± 4 ^†^
	CON	29 ± 3	26 ± 3	23 ± 3 *	24 ± 4	26 ± 4

Values are means ± SE. *: *p* < 0.05 vs. pre, ^†^: *p* < 0.05 vs. CON.

**Table 2:** Change in blood variables.

		Pre	Post	
			0 min	30 min
Glucose (mmol/L)	EX	4.92 ± 0.05 ^†^	5.30 ± 0.12 *^,†^	4.64 ± 0.08 *^,†^
	CON	4.78 ± 0.05	4.84 ± 0.05	4.93 ± 0.04 *
Lactate (mmol/L)	EX	1.6 ± 0.2	1.6 ± 0.2	1.6 ± 0.1
	CON	1.4 ± 0.1	1.5 ± 0.1	1.4 ± 0.1
GH (ng/mL)	EX	1.8 ± 0.5	8.9 ± 1.8 *^,†^	4.1 ± 0.8 ^†^
	CON	2.5 ± 0.6	2.1 ± 0.4	1.3 ± 0.3
FFA (mmol/L)	EX	0.42 ± 0.05	1.22 ± 0.08 *^,†^	0.90 ± 0.08 *^,†^
	CON	0.38 ± 0.03	0.35 ± 0.03	0.55 ± 0.04 *
Mb (ng/mL)	EX	36 ± 2	136 ± 26 *^,†^	140 ± 22 *^,†^
	CON	37 ± 3	36 ± 2	35 ± 2
CK	EX	349 ± 27	457 ± 30 *	436 ± 29 *
(U/L)	CON	402 ± 74	389 ± 68 *	385 ± 70 *

Values are means ± SE. *: *p* < 0.05 vs. pre, ^†^: *p* < 0.05 vs. CON.

**Table 3:** Energy intake, macronutrient intake ratio and categories of selected menus.

		EX	CON
**General information**			
Duration of eating	(min)	22 ± 1	23 ± 1
Energy intake	(kcal)	1325 ± 55 ^†^	1529 ± 55
**Detailed information**			
Macronutrient intake			
Protein	(%)	14.6 ± 0.5	15.3 ± 0.5
	(g)	49 ± 3 ^†^	58 ± 2
Fat	(%)	26.2 ± 1.4 ^†^	29.8 ± 1.2
	(g)	39 ± 3	51 ± 3
Carbohydrate	(%)	59.2 ± 1.9 ^†^	54.9 ± 1.5
	(g)	190 ± 9	202 ± 10
Categories of selected menus			
Staple food (rice and bread)	(kcal)	545 ± 34 ^†^	659 ± 45
Others	(kcal)	553 ± 38 ^†^	694 ± 28
Fruits	(kcal)	101 ± 15	75 ± 12
Drinks (tea, juice milk and soup)	(kcal)	126 ± 11 ^†^	101 ± 16

Values are means ± SE. ^†^: *p* < 0.05 vs. CON.

## Abbreviations

PYY	peptide YY
GLP-1	glucagon-like peptide-1
$\dot{V}$O_2max_	maximal oxygen uptake
HIIT	high-intensity interval training
VAS	visual analog scale
GH	growth hormone
FFA	free fatty acid
Mb	myoglobin
CK	creatine kinase
CV	coefficients of variation
IL-6	interleukin-6
